# Roles for the RNA polymerase III regulator MAFR-1 in regulating sperm quality in *Caenorhabditis elegans*

**DOI:** 10.1038/s41598-020-76423-5

**Published:** 2020-11-09

**Authors:** Amy M. Hammerquist, Sean P. Curran

**Affiliations:** 1 Leonard Davis School of Gerontology, University of Southern California, Los Angeles, CA USA; 2 Department of Molecular and Computational Biology, Dornsife College of Letters, Arts, and Science, University of Southern California, Los Angeles, CA USA; 3 Norris Comprehensive Cancer Center, University of Southern California, Los Angeles, CA USA

**Keywords:** Transcription factors, Non-coding RNAs, Transcription, Transcriptional regulatory elements

## Abstract

The negative regulator of RNA polymerase (pol) III *mafr-1* has been shown to affect RNA pol III transcript abundance, lipid biosynthesis and storage, progeny output, and lifespan. We deleted *mafr-1* from the *Caenorhabditis elegans* genome and found that animals lacking *mafr-1* replicated many phenotypes from previous RNAi-based studies and discovered a new sperm-specific role. Utilizing a yeast two-hybrid assay, we discovered several novel interactors of MAFR-1 that are expressed in a sperm- and germline-enriched manner. In support of a role for MAFR-1 in the male germline, we found *mafr-1* null males have smaller spermatids that are less capable in competition for fertilization; a phenotype that was dependent on RNA pol III activity. Restoration of MAFR-1 expression specifically in the germline rescued the spermatid-related phenotypes, suggesting a cell autonomous role for MAFR-1 in nematode male fertility. Based on the high degree of conservation of Maf1 activity across species, our study may inform similar roles for Maf1 and RNA pol III in mammalian male fertility.

## Introduction

Canonically characterized as a negative regulator of RNA polymerase (pol) III, *MAF1* was originally discovered and has been extensively studied in *Saccharomyces cerevisiae*^1–3^. Since its discovery, *MAF1* has been identified across diverse eukaryotic clades^2–7^. Perturbation of *MAF1* activity leads to, in addition to increased RNA pol III activity, an increase in intracellular lipid abundance^8–10^ and in some instances altered lifespan^11,12^. The majority of studies of *MAF1* have been conducted in single-cell systems (as *S. cerevisiae*) or cultured mammalian cells^4,10,13,14^, but relatively few studies have probed the function of *MAF1* within the context of a complete animal^8,15–17^. In *Caenorhabditis elegans*, RNA interference (RNAi) knockdown of *MAF1* homolog *mafr-1* results in increased RNA pol III activity, increased intestinal lipid accumulation (as well as increased expression of lipid biogenesis genes), and increased expression of the vitellogenin family of lipid transport proteins^8^. As vitellogenesis is the process by which lipids are transferred to developing oocytes, these findings imply a role for MAFR-1 in reproduction, but this has not yet been fully investigated.

Although RNA pol III activity has, to our knowledge, not been directly implicated in fertility, many processes affected by RNA pol III activity do affect reproductive output. *Drosophila* with disrupted ribosome biogenesis display a Minute phenotype characterized by delayed development, short and thin bristles, and impaired fertility and viability^18^. Furthermore, mice lacking *Zfn384*, a protein whose sub-cellular location was highly correlated with that of RNA pol III in human embryonic stem cells, have fertility defects and impaired spermatogenesis^19,20^. While mature sperm are widely accepted to be transcriptionally and translationally quiescent^21,22^, as a repressor of RNA pol III activity, MAFR-1 may still be acting in developing germ cells to prevent erroneous RNA pol III transcription.

In all sexually reproducing species, sperm are produced in great excess of oocytes, and must compete with each other to successfully fertilize an oocyte. Generally, larger sperm are able to travel faster than smaller sperm, and therefore have a competitive advantage^23–25^. As a hermaphroditic species, *C. elegans* presents a unique situation: the sperm produced by hermaphrodites are capable of fertilizing oocytes but are almost completely outcompeted by male sperm, when mated^26^. In this work, we explore the role of MAFR-1 in maintaining male sperm quality in *C. elegans*, which affects sperm quality metrics such as size and activation capacity, as well as competitive advantage over hermaphrodite sperm.

## Results

### Characterization of a *mafr-1* null mutant

**Figure 1:**
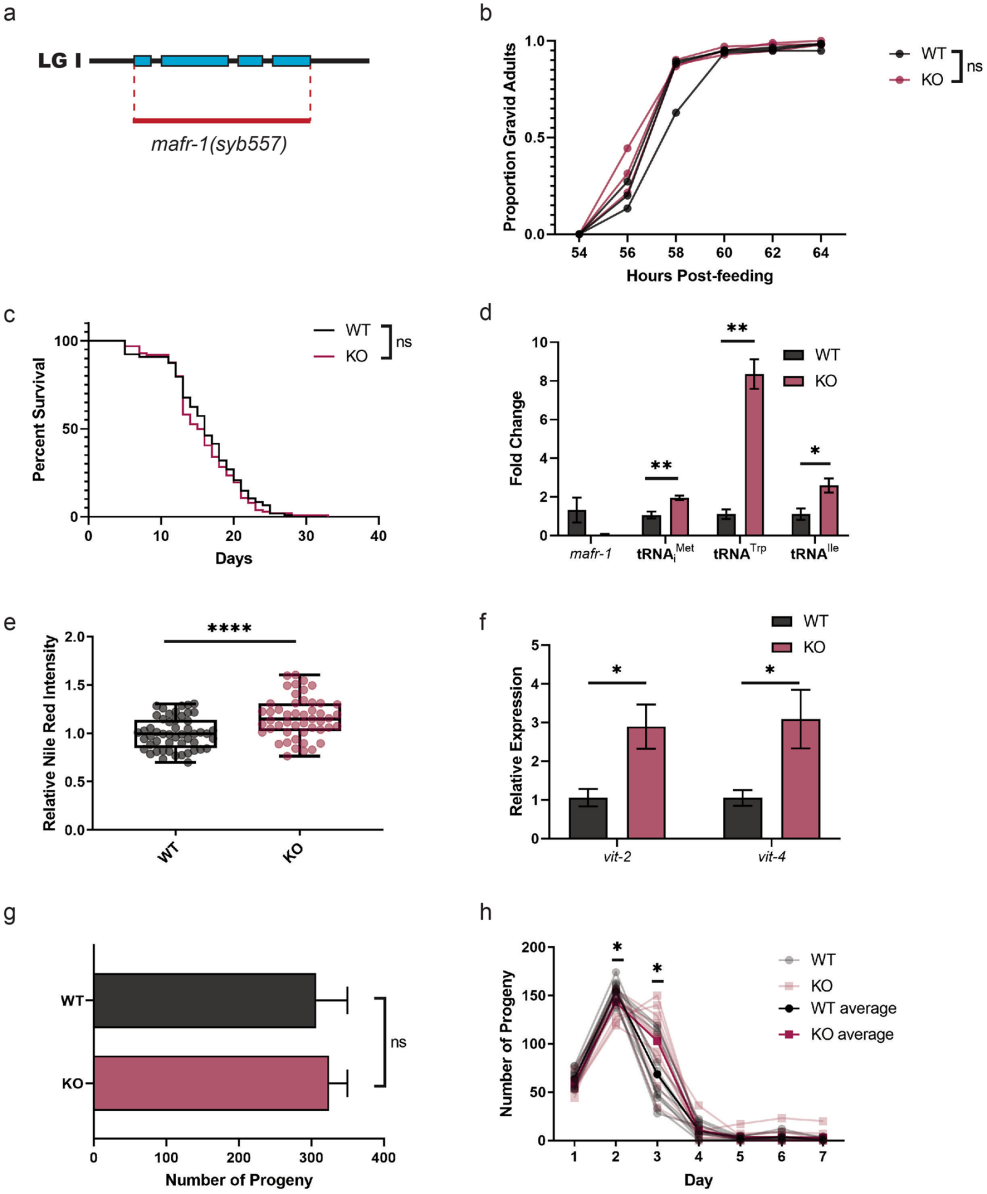
*mafr-1* (KO) produces fertile, viable hermaphrodites. (**a**) Schematic diagram showing region deleted in *mafr-1(syb557)*, a CRISPR-generated and sequence confirmed deletion of the entire *mafr-1/C43H8.2* coding sequence from the start to stop codons, hereafter referred to as *mafr-1* (KO) (Adobe Illustrator v24.3). (**b**) Developmental time course of WT and *mafr-1* (KO) hermaphrodites. Each line represents one population of > 100 animals. (**c**) Lifespan survival assay of WT and *mafr-1* (KO) hermaphrodites. Individual lines represent average of two or three biological replicates of 50-worm populations (see Fig. S1a). No significant difference was found by Log-rank (Mantel-Cox) test. (**d**) Quantitative PCR expression analysis of *mafr-1* and three tRNA RNA pol III transcripts: initiator Methionine, Tryptophan, and Iso-leucine. (**e**) Lipid abundance as measured by Nile Red staining intensity. (**f**) Quantitative PCR expression of *vit-2* and *vit-4*. (**g**) Total brood size of WT and *mafr-1* (KO) hermaphrodites (n > 10). (**h**) Time course showing reproductive output of WT and *mafr-1* (KO) hermaphrodites throughout reproductive span. Unless otherwise specified, statistical comparisons made by Student’s t-test (two-tailed). ns = no significance, *p < 0.05, **p < 0.01, ***p < 0.001, ****p < 0.0001.

Previous studies of *mafr-1* in *C. elegans* have utilized RNAi-based approaches^8^ and a gain-of-function allele of *mafr-1*^16^ leaving the true loss-of-function phenotype unknown. To better examine the biological functions of MAFR-1, we assessed the impact of a true molecular null allele of *mafr-1, a* CRISPR-generated and sequence confirmed deletion of the entire *mafr-1/C43H8.2* coding sequence from start to stop, hereafter referred to as *mafr-1* (KO) (Fig. 1a). Surprisingly, a total loss of *mafr-1* did not result in changes in developmental timing (Fig. 1b) or overall organismal health, as *mafr-1* (KO) animals had similar lifespans to wild type (WT) controls (Fig. 1c*,* Fig. S1a). While *mafr-1* expression was undetectable in *mafr-1* (KO) animals (Fig. 1d), expression of genes downstream in the CEOP1628 operon, *arch-1* and *B0511.6*, were unaffected (Fig S1b). Consistent with its canonical role as a negative regulator of RNA pol III activity and previously observed phenotypes for *mafr-1* RNAi^2,3,8^, *mafr-1* (KO) animals showed increased expression of RNA pol III transcripts, including three tRNAs: initiator Methionine, Tryptophan, and Iso-leucine (Fig. 1d). Similarly, *mafr-1* (KO) animals displayed increased intracellular lipid abundance relative to age-matched WT control animals (Fig. 1e), as expected from previous studies^8–10,16^. Additionally, the expression of vitellogenins, which deliver lipids from the intestine to the germline to drive reproduction, was previously demonstrated to increase in *mafr-1* RNAi treated animals and diminished by *mafr-1* overexpression^8^, and was increased *mafr-1* (KO) animals relative to WT controls at the transcriptional (Fig. 1f), but not the protein, level (Fig. S1c). Our previous investigations revealed that overexpression of *mafr-1* can influence reproductive output^8^. Although the total reproductive output between *mafr-1* (KO) animals and WT controls were not significantly different (Fig. 1g), peak reproductive output appeared delayed in *mafr-1* (KO) animals (Fig. 1h). Taken together, these results indicate that *mafr-1* (KO) animals share several of the previously documented *mafr-1*-associated phenotypes, without compromised overall health.

### MAFR-1 interacts with sperm-enriched proteins

**Figure 2:**
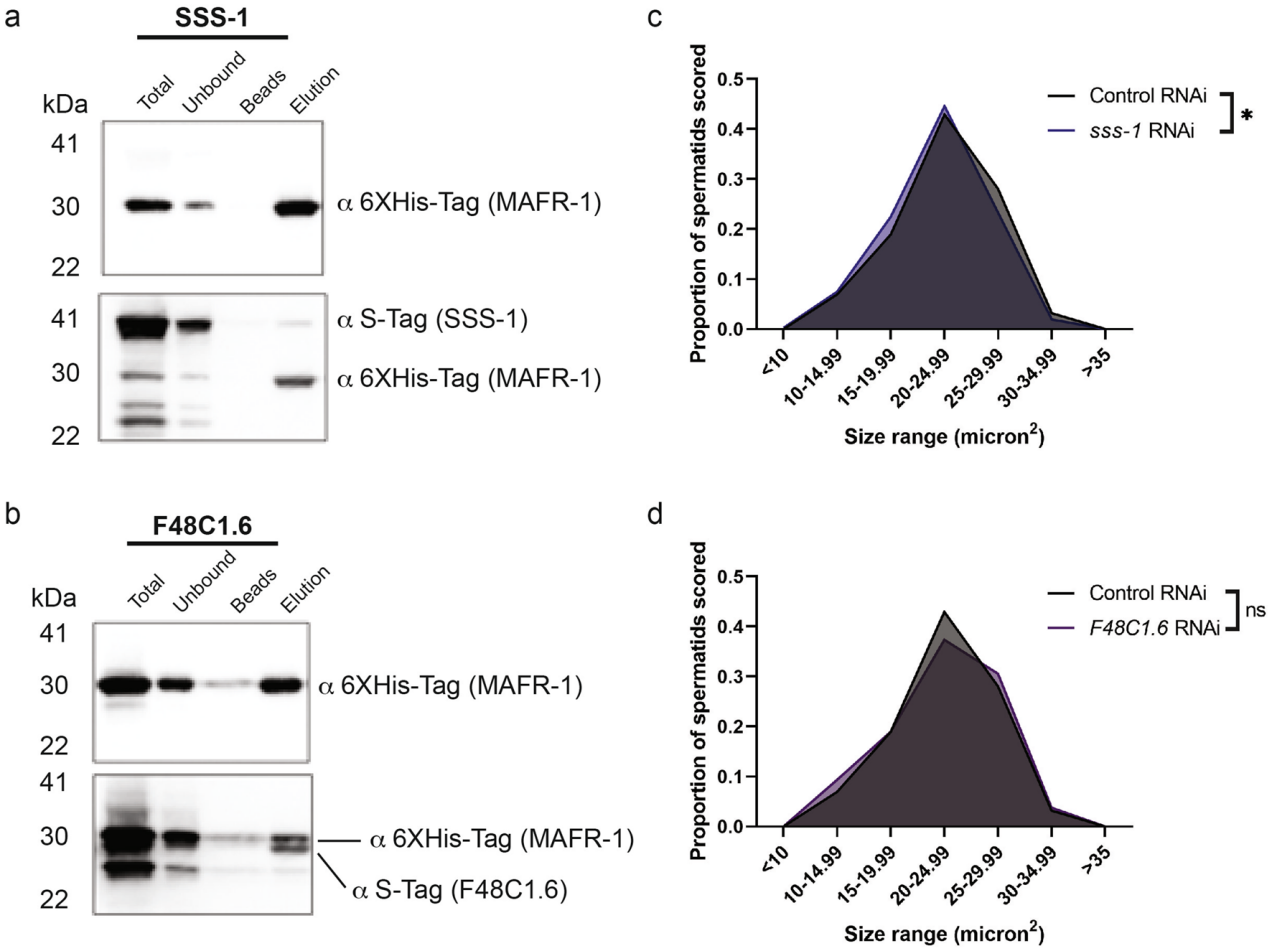
MAFR-1 interacts with SSS-1 and F48C1.6, which affect spermatid size. (**a**,**b**) Western Blot analysis of Ni–NTA column co-purification of MAFR-1 and SSS-1 (**a**), and MAFR-1 and F48C1.6 (**b**). (**c**,**d**) Spermatid size in day 1 adult males following RNAi depletion of *sss-1* (**c**), and *F48C1.6* (**d**)*.* See Supplemental Data Set for further statistical analysis. The same Control RNAi data are depicted in (**c**,**d**). Experiments done in biological triplicate, statistical comparisons made by Student’s t-test (two-tailed). ns = no significance, *p < 0.05, **p < 0.01, ***p < 0.001, ****p < 0.0001.

MAF1 has been shown to physically interact with components of the RNA pol III complex^27–30^, but other direct interactors remain elusive. In order to look for novel protein interactors with MAFR-1, we performed a yeast two-hybrid screen using MAFR-1 as bait. We identified 62 putative protein–protein interactors of MAFR-1 (Table S1), which represent GO-terms comprising multiple essential cellular processes (Fig. S2a). Among these hits, we defined a novel class of germline- or spermatid-enriched^31^ putative MAFR-1 interactors: SSS-1, F48C1.6, and MSP-53. Because a role for MAF1 in germ cells has not been previously described, we chose these putative interactors for further analysis. We first examined the RNA expression levels of each putative interactor in wild type and *mafr-1* (KO) animals by qPCR, which revealed increased expression of *sss-1* and *msp-53*, but not *F48C1.6* relative to WT (Fig. S1b). Next, we confirmed the physical interaction of MAFR-1 with SSS-1 (Fig. 2a), and F48C1.6 (Fig. 2b) biochemically by co-expression in *E.coli* followed by affinity purification MAFR-1, which facilitated the co-purification of each interactor (Fig. 2a,b). While we observed clear enrichment for SSS-1 and F48C1.6, our ability to measure enrichment of MSP-53 was confounded by its association with the Ni–NTA resin (Fig. S2c); a quality likely associated with its known capacity to mediate multiple interactions^32,33^. Based on their enriched expression in spermatids, we next assessed the impact of reducing the expression of SSS-1 or F48C1.6 by RNAi on sperm quality. Sperm size is a well-established quality that influence male reproductive success through sperm competition^24^. *Caenorhabditis elegans* males produce sperm that are significantly larger than hermaphrodite sperm and this size difference facilitates male competitive advantage when mating occurs. As expected based on their enrichment in germ cells, RNAi of *sss-1* and *msp-53*, but not *F48C1.6*, resulted in decreased spermatid size (Fig. 2c,d, Fig. S2d-e).

In addition to size, the speed of male sperm is greater than hermaphrodite-derived sperm, which also enhances male competitive advantage for fertilizing an oocyte^24^. To become motile, inactive spermatids must become activated by developing pseudopodia, a sophisticated process under the control of genetic and environmental factors^32,34,35^. In the laboratory, isolated spermatids can be activated in vitro by exposure to Pronase^34,35^. RNAi of these potential interactors resulted in no significant change in activation (Fig. S2f); however, as several validated Spe genes are not sterile when inactivated by RNAi^36,37^, future studies of the role of MAFR-1 and sperm-enriched proteins is warranted. Nevertheless, in light of these findings, we hypothesized that MAFR-1 might also function in the male germline to influence reproductive success.

### MAFR-1 regulates male sperm fitness

**Figure 3:**
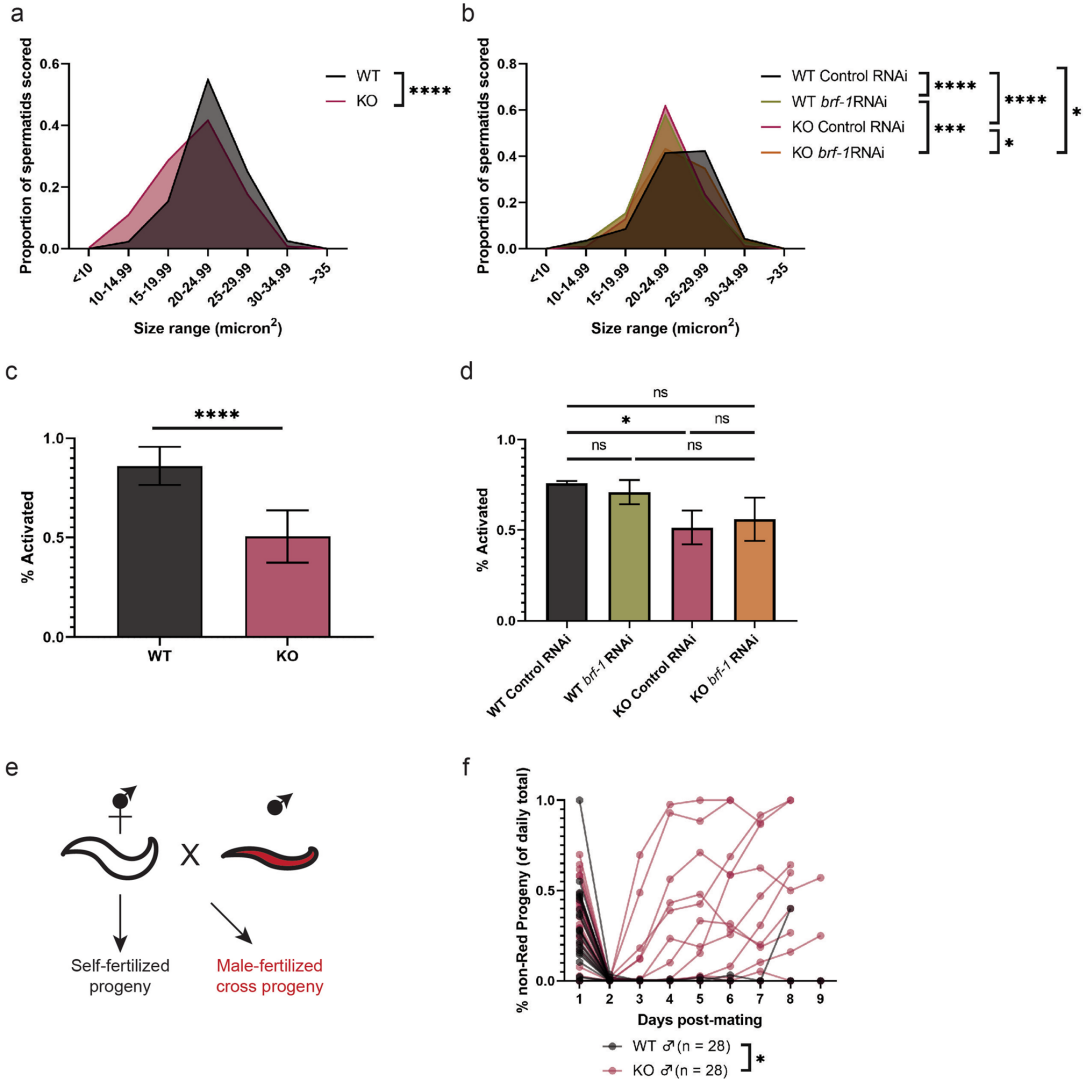
*mafr-1* (KO) males have diminished sperm quality. (**a**) Spermatid size in WT and *mafr-1* (KO) males. See Supplemental Data Set for further statistical analysis. (**b**) Spermatid size of day 2 adult WT and *mafr-1* (KO) males following RNAi knockdown of *brf-1*. See Supplemental Data Set for further statistical analysis. (**c**) In vitro Pronase activation of WT and *mafr-1* (KO) male spermatids. (**d**) In vitro Pronase activation of day 2 adult WT and *mafr-1* (KO) males following RNAi knockdown of *brf-1*. (**e**) Schematic diagram showing setup of male sperm competition mating assay (Adobe Illustrator v24.3). (**f**) Proportion of progeny fertilized by self-sperm from WT- and *mafr-1* (KO)-mated hermaphrodites. Experiments done in biological triplicate. Statistical comparisons made by Student’s t-test (two-tailed). ns = no significance, *p < 0.05, **p < 0.01, ***p < 0.001, ****p < 0.0001.

To investigate the role of MAFR-1 in spermatogenesis, we looked at spermatid size and activation. We found that *mafr-1* (KO) males produced significantly smaller spermatids (Fig. 3a*,* Fig. S3a). Interestingly, while some physiological responses, including reproduction, are sensitive to diet type^8,16,35,38–43^, the reduced size phenotype in *mafr-1* (KO) males is also observed on the HT115 *E.coli* K-12 bacterial diet (Fig. S3b-c). Next, we sought to determine the causal relationship between MAFR-1 negative regulation of RNA pol III activity and spermatid size. BRF-1 is a transcription factor required for proper RNA pol III activity^44–48^. Because *mafr-1* (KO) animals have increased RNA pol III activity, we used RNAi against *brf-1* and found that male spermatid size was restored in *mafr-1* (KO) animals (Fig. 3b, Fig. S3d). These results suggest a role for MAFR-1 in regulating sperm quality and competitive ability.

We also found that *mafr-1* (KO) male sperm are less capable of activation upon treatment with Pronase (Fig. 3c). Thus, *mafr-1* (KO) males have smaller sperm with a reduced capacity to mature, which could impact their ability to fertilize hermaphrodite oocytes. Interestingly, we found that RNAi knockdown of *brf-1* did not significantly affect spermatid activation in WT or *mafr-1* (KO) animals (Fig. 3d).

In order to test the physiological consequence of *mafr-1* (KO) on sperm fitness phenotypes, we designed an assay to assess sperm quality^35^. When hermaphrodites are mated to WT males, nearly all resulting progeny are derived from male sperm^26^. This can be visualized if males harboring a wrmScarlet transgene are used for mating; progeny derived from male sperm express wrmScarlet while progeny stemming from hermaphrodite self-sperm do not express wrmScarlet (Fig. 3e). Thus, the proportion of non-Red progeny corresponds to utilization of self-sperm. When WT males expressing a CRISPR-integrated, single-copy, ubiquitously-expressed wrmScarlet marker were mated to WT hermaphrodites, nearly all subsequent progeny over the reproductive span expressed wrmScarlet, and were therefore derived from male sperm acquired through mating (Fig. 3f). In contrast, when wrmScarlet-expressing *mafr-1* (KO) males were mated to WT hermaphrodites, 10 of 28 individuals produced progeny that were non-Red (as opposed to 1 of 28 in WT males), which indicates a significant proportion of self-sperm-fertilized progeny, and implies impairment of these *mafr-1* (KO) male sperm to outcompete WT hermaphrodite self-sperm. Taken together, these data reveal a novel role for MAFR-1 in spermiogenesis.

### MAFR-1 impacts sperm quality cell autonomously

**Figure 4:**
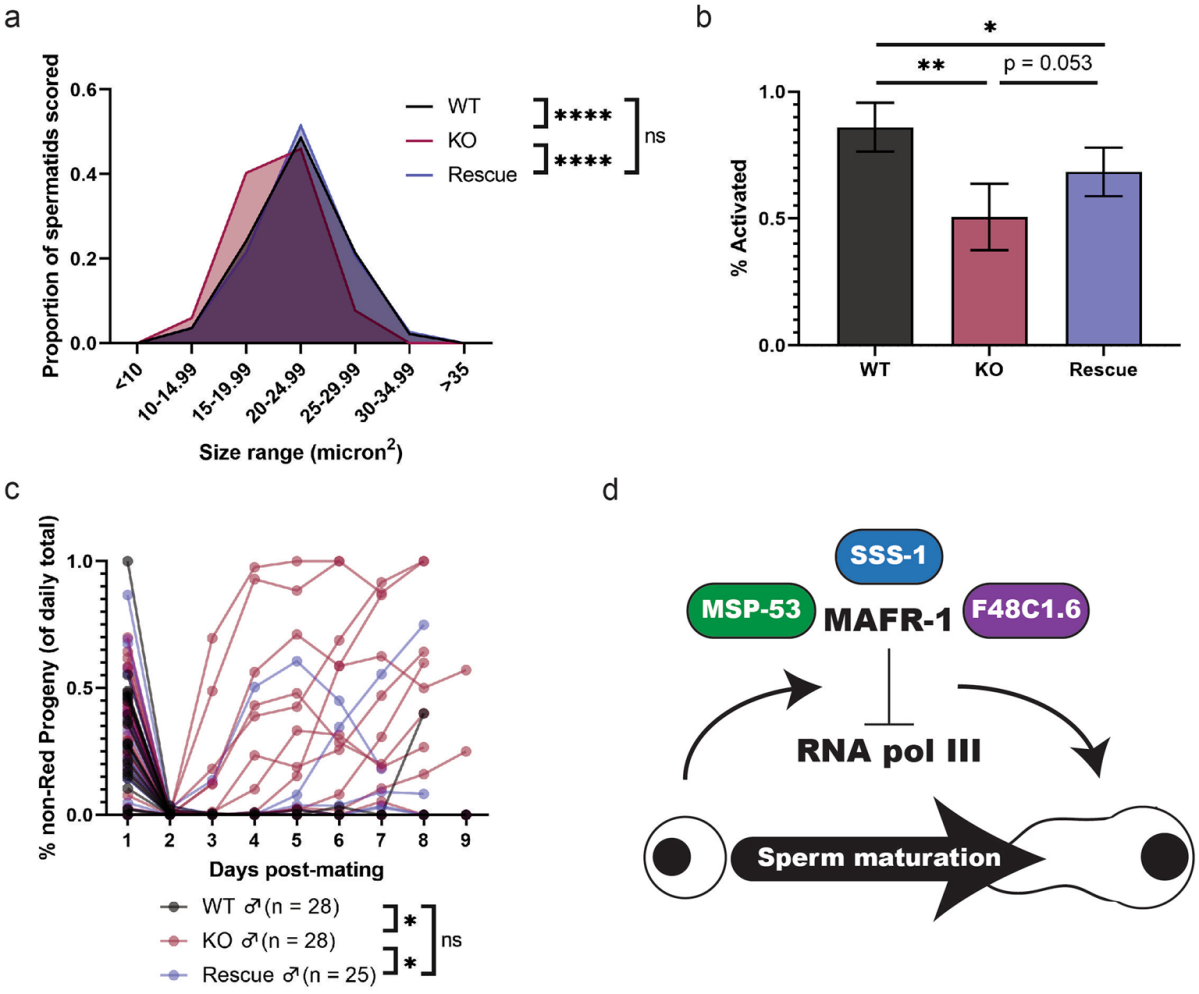
Germline expression of WT *mafr-1* rescues sperm quality phenotypes. (**a**) Spermatid size in WT, *mafr-1* (KO), and germline-specific rescue males. See Supplemental Data Set for further statistical analysis. (**b**) In vitro Pronase activation of WT, *mafr-1* (KO), and germline-specific rescue male spermatids. *mafr-1* (KO)/Rescue comparison done via unpaired, one-tailed t-test. Experiments done in biological triplicate. (**c**) Proportion of progeny fertilized by self-sperm from WT, *mafr-1* (KO), and germline-specific rescue-mated hermaphrodites. WT and *mafr-1* (KO) data are same data presented in Fig. 3e. (**d**) Model of effects of MAFR-1 on spermatid quality. MAFR-1 interacts with MSP-53, SSS-1, and F48C1.6, and its repression of RNA pol III is required for proper sperm maturation (Adobe Illustrator v24.3). Unless otherwise specified, statistical comparisons made by Student’s t-test (two-tailed). ns = no significance, *p < 0.05, **p < 0.01, ***p < 0.001, ****p < 0.0001.

One possible explanation for the discrepancy of phenotypes resulting from RNAi and genetic studies, is the lack of uniformity of RNAi across different tissues^49–53^. To assess whether MAFR-1 functions cell autonomously in the germline to regulate sperm quality, we restored MAFR-1 in the germline of *mafr-1* (KO) animals, driving *mafr-1* expression with the *pie-1* promoter^54–56^ (Fig. S4a). Animals with germline-specific rescue of *mafr-1* developed normally into fertile adults, but exhibited a slight developmental delay (Fig. S4b). Germline-specific expression of *mafr-1* in *mafr-1* (KO) males restored spermatid size to that of WT males (Fig. 4a, Fig. S4c), while partially restoring spermatid activation (Fig. 4b). Importantly, germline rescue of MAFR-1 also restored the competitive ability of *mafr-1*(KO) male sperm in our mating assay, with only 2 of 25 Rescue-mated WT hermaphrodites producing non-Red progeny (Fig. 4c), without affecting total brood size (Fig. S4d). These data suggest a cell-autonomous role for MAFR-1 in the male germline and collectively our study reveals a role for MAFR-1 in multiple parameters of sperm quality and male reproductive fitness (Fig. 4d).

## Discussion

In light of the conflicting phenotypes associated with the loss of *MAF1* by RNA interference (RNAi), genetic mutations in metazoans^8,15–17,43^, and in cultured cell models^10^, we characterized a loss-of-function allele in a second metazoan model. *mafr-1* (KO) *C. elegans* display no gross defects– males have no obvious structural changes to the male copulatory organ when compared to WT (Fig. S3e-f) and are generally healthy (Fig. 1b,c), with brood sizes comparable to WT (Fig. 1g). Nevertheless, the novel sperm-enriched interactors discovered in a yeast two-hybrid screen (Fig. 2, Fig. S2) prompted us to investigate the roles of MAFR-1 in the germline, specifically of males, which had not been previously studied. Recent work has found MAF1 expression in embryonic stem cells drives differentiation^13^. *Caenorhabditis elegans* possess only one population of stem cells—the germline—which originate from a single cell and become progressively more mature as they migrate through the gonad away from the distal tip cell^57^. In fact, previous studies have found *mafr-1* enriched in the *C. elegans* germline, but the significance of this finding has never been studied^58–60^.

While previous work documented altered vitellogenin expression at both the transcriptional and protein level in response to *mafr-1* RNAi^8^, we observed altered vitellogenin expression only at the mRNA level (Fig. 1f, Fig. S1c). This discrepancy could be explained by post-transcriptional regulatory pathways that govern intestinal synthesis of the vitellognenins, export from intestinal cells, or endocytosis in oocytes. Additionally, it is possible that *mafr-1* RNAi results in different regulation of vitellogenins through persistent expression, as RNAi provides significant knockdown, but not complete ablation, of gene expression. RNAi is not uniformly effective across tissues, providing inefficient knockdown in the neurons^51,52^, pharynx^53^, and vulva^49,61^, which could be acting cell non-autonomously to cause pleiotropic gene expression phenotypes. As reproduction was generally normal in *mafr-1* (KO) animals, it is not surprising that other regulatory mechanisms might restore homeostasis. Nevertheless, our studies in males, which do not express vitellogenins, identifies a novel role of *mafr-1* in proper sperm function.

Furthermore, it is likely that regulation of vitellogenin expression may be sensitive to diet, as *mafr-1* expression and *mafr-1*-sensitive phenotypes are altered on an *Escherichia coli* K12/HT115 diet, in which the vast majority of RNAi studies are performed, relative to the standard *E. coli* B/OP50 diet^8^. Similarly, spermatid size appears to be sensitive to diet, with HT115-fed *mafr-1* (KO) males producing significantly smaller sperm than those reared on OP50 (Fig. S3b-c). MAFR-1 plays an important role coordinating regulation of biosynthetic capacity in response to stimuli from the insulin signaling pathway^8,10,17,62,63^, which may be induced on the HT115 diet, as endogenous carbohydrates are 3- to 5-fold higher in HT115 relative to OP50^64^.

Prior to this study, known physical interactors of MAF1 and its homologs were limited to regulatory kinases and phosphatases^65–67^, as well as RNA pol III subunits and transcription factors^27–30^. Although the molecular function of this interaction is not fully understood, the MSP family of proteins play multiple roles in sperm function and biogenesis^32,33^. Interaction of MAFR-1 with proteins expressed in a tissue-specific manner implies that MAFR-1 could play additional roles that are unique to distinct cell types. Furthermore, restoration of WT *mafr-1* expression in the germline partially rescued all measured metrics of sperm quality (Fig. 4a–c), indicating the cell-autonomous nature of the role of MAFR-1 in sperm maturation. To the best of our knowledge, this is the first instance of manipulation of any MAF1 homolog in any individual tissue of a multicellular organism. Taken together, our findings suggest that MAF1 may have unique roles in unique tissues that warrant further investigation.

While *mafr-1* (KO) animals have similar lifespans (Fig. 1c) and developmental rates (Fig. 1b) to WT, germline-rescued *mafr-1* (KO) animals take approximately two hours longer than WT or *mafr-1* (KO) to become gravid adults (Fig. S3b). MAF1 is activated in human cells in response to serum starvation^68,69^, and confers starvation resistance in other organisms^3,6^. Expression of MAFR-1 under a non-endogenous promoter prevents it from being transcriptionally regulated by its normal cues, which could conceivably result in perceived starvation. In the germline, *C. elegans* have been shown to slow oogenesis in response to starvation^70^, and thus, *pie-1* promoter-driven MAFR-1 expression may cause the observed delay in reproductive maturation of *mafr-1* (KO) animals through perceived starvation in the germline.

MAF1 is generally viewed as a repressor of growth^63^. Seemingly contrary to this paradigm, spermatids from males lacking *mafr-1* are smaller than those of WT males (Fig. 3a). In *C. elegans*, as sperm size is correlated with competitive advantage, and therefore general sperm quality^24^, small sperm can be an indication of poor sperm quality. RNA pol III transcripts make up an significant fraction of spermatid RNA populations^71,72^, and while mature spermatids are transcriptionally and translationally quiescent^4,22^, erroneous RNA pol III activity in germ cells likely results in lower quality, and therefore smaller, spermatids. Rescue of MAFR-1 expression in the germline rescued the diminished spermatid size of *mafr-1* (KO) males, which suggests *mafr-1* activity is required in a cell-specific manner for spermatid size (Fig. 4a). In addition, RNAi of the RNA pol III transcription factor *brf-1* restored spermatid size revealing that this phenotype is linked to aberrant RNA pol III activity (Fig. 3b). Similarly, sperm activation was rescued by restoration of WT MAFR-1 in the germline (Fig. 4b), but the effects of *brf-1* RNAi on activation were not significant. Based on the effect of *brf-1* RNAi in WT spermatids, it appears that the loss of RNA pol III activity is perhaps pleiotropic, potentially because of the role of proteostasis in sperm activation^34,35^. Nevertheless, the trend was toward restoring sperm activation defects stemming from loss of *mafr-1* when *brf-1* is reduced by RNAi (Fig. 3d). Our results suggest that tight regulation of RNA pol III activity, through MAFR-1, is important for proper sperm function.

In all instances we employed RNAi to explore the role of a gene in regulating sperm quality, we found significant effects on sperm size but not spermatid activation (Figs. 2c,d, 3b,d, Fig. S2d-f). While this suggests that sperm size is the more sensitive assay, these results do not necessarily indicate that the genes investigated do not play a role in sperm maturation. Male gametes appear to be another tissue in which RNAi efficiency is reduced^36,37^, as several sperm-specific sterility phenotypes caused by genetic mutants cannot be recapitulated through RNAi^37^. Genetic manipulation is necessary to determine the role of these genes in spermiogenesis, but a lack of viable mutants leaves RNAi as the best currently available tool to study *sss-1*, *F48C1.6*, *msp-53*, and *brf-1* in this context. We note that our discovery of the sperm-specific phenotypes of *mafr-1* might not have been uncovered without the development of the CRISPR-generated null allele. In summary, our work defines three novel interactors of MAFR-1, and a unique role in the male germline affecting sperm maturation, likely through its regulation of RNA pol III activity (Fig. 4d).

## Experimental procedures

### *Caenorhabditis elegans* strains and maintenance

*Caenorhabditis elegans* were maintained at 20 °C on 6 cm plates of Nematode Growth Medium (NGM) supplemented with streptomycin and seeded with OP50-1 *E. coli*. For RNAi experiments, NGM plates were supplemented with 5 mM IPTG and 100 μg/ml carbenicillin and seeded with HT115 *E. coli* expressing dsRNA targeting gene as specified.

The following strains were used: wild type (WT) N2 Bristol, PHX557[*mafr-1(syb557)* I], SPC489 [*mafr-1(syb557)* I;* laxSi01– pie-1p::mafr-1::mafr-1 3′UTR cb-unc-119(* +*)*II], WBM1143 [*wbmIs67–eef-1A.1p::3XFLAG::wrmScarlet::unc-54 3′UTR*V], SPC490 [*mafr-1(syb557)* I*; wbmIs67–eft-3p::3XFLAG::wrmScarlet::unc-54 3′UTR*V], SPC491 [*mafr-1(syb557)* I;* laxSi01– pie-1p::mafr-1::mafr-1 3′UTR cb-unc-119(* +*)*II;* wbmIs67—eft-3p::3XFLAG::wrmScarlet::unc-54 3UTR*V], DH1033 [*sqt-1(sc103)* II;* bIs1 – vit-2::GFP rol-6(su1006)* X], SPC492 [*mafr-1(syb557)* I;* sqt-1(sc103)* II;* bIs1 – vit-2::GFP rol-6(su1006)* X].

### Yeast-2-hybrid screen

Bait and prey plasmids were generated by cloning *mafr-1* into pLexA and a *C. elegans* cDNA library into pACT2.2 (Addgene), respectively. Interaction was tested on synthetic complete agar that lacked leucine and tryptophan and was supplemented with X-α-gal. Interactors were identified by transforming Y2HGold (Clontech) with bait (pLexA-MAFR-1) and the cDNA prey library. Positive clones were grown in the absence of tryptophan and sequenced. Sequenced clones were then retested individually.

### Biochemical co-purification

MAFR-1 and respective interactors were cloned into MCS1 and MCS2 of pCOLA2, respectively. Origami K-12 *E. coli* were grown to stationary phase and expression of pCOLA2 vector was induced using IPTG for 2 h. Cells were pelleted from 50 ml induced culture, and frozen. Proteins were isolated and purified as in Ni–NTA Purification System protocol (Qiagen).

### RNA extraction and gene expression

Worms were washed from plates using M9 containing 0.01% Triton-X100, washed twice in M9, and frozen at − 80 °C in 500 μl TRI-Reagent (Zymo). Frozen worms were thawed on ice, and cuticles manually disrupted using 25G needle. RNA was extracted using Direct-zol RNA Miniprep kit (Zymo Research R2071). cDNA was synthesized using qScript reverse transcription kit (Quanta Biosciences), diluted, and quantitative PCR was performed using PerfecTA SYBR Green (Quanta Biosciences). All genes were normalized to expression of *snb-1*. Primers used as previously described in Pradhan et al. and Khanna et al.^16^. New primers used in this study are as follows:

**Table:** 

Gene name	Forward primer sequence	Reverse primer sequence
*msp-53*	TGCCATAATCTTCACTGCGAG	TCCTTTGGGTCGAGAACTCC
*sss-1*	CTACGGCATCACTTTTCGGG	GAAACAGATTGAGCCGTGCA
*F48C1.6*	GGCGTAGTTCCTTTAGTGCG	AGCTCGTGGAGATTGTTGGA

### Nile red lipid staining

Synchronous L4 animals were stained as in^73^. Briefly, animals were washed from plate, fixed in 40% isopropanol for 3 min, stained in 0.03 mg/ml Nile Red in 40% isopropanol for 2 h, and de-stained in PBS with 0.01% Triton for 30 min. Stained worms were mounted in de-staining solution and imaged using DIC and GFP filters on Zeiss Axio Imager, with ZEN software. Fluorescence of individual worms was measured using ImageJ software (NIH).

### Lifespan analysis

Lifespan data was collected as previously described^40,74–76^. In brief, plates containing synchronized populations were scored daily for survival, beginning at L4 stage. Animals were moved to new plates periodically during the reproductive span to remove progeny. Each line represents the average of two (in the case of WT) or three (in the case of *mafr-1* (KO)) biological replicates of 50 animals each. Animals that died of bursting, matricide, or crawling off plate were censored.

### Reproduction

Self-reproduction: L1-synchronized hermaphrodites were grown to L4 and single worms were placed on individual plates. Worms were transferred to fresh plates every 24 h until egg laying ceased, and progeny were counted 48 h after hermaphrodite was removed from plate.

Mated-reproduction: Single L4 hermaphrodites were placed with virgin day 1 adult males (harboring a CRISPR-integrated, single-copy, *eef-1A.1* promoter-driven wrmScarlet transgene) in a 1:1 ratio on plates seeded with 20 μl OP50, and allowed to mate overnight. Males were removed from plates, and hermaphrodites were transferred to fresh plates every 24 h until egg laying ceased. Progeny were counted and scored for wrmScarlet fluorescence 48 h after hermaphrodite was moved from plate. Animals were censored as “not sufficiently mated” if male sperm did not suppress self-progeny production to < 5% of the daily total on day 2. Hermaphrodites were counted as producing non-Red progeny if > 2% of the total progeny produced after day 1 of adulthood were non-Red (progeny produced on the first day were not included due to variance in mating efficiency).

### Sperm size analysis

Spermatids were isolated as previously described^35^. In brief, five virgin day 1 adult males were dissected in SM buffer containing dextrose to release spermatids. Spermatids were imaged using Zeiss Axio Imager, and size measured using ImageJ software. Unless otherwise indicated, unique WT controls were performed for each figure panel. For experiments in which *sss-1*, *F48C1.6*, and *msp-53* RNAi were employed, animals were hatched on RNAi, and treated as OP50 animals—L4s were sequestered for 24 h and virgin day 1 adult males were dissected. Dissected gonads that released activated and mature sperm, instead of only spermatids were censored.

For experiments in which *brf-1* RNAi was employed, RNAi was administered post-developmentally: animals were hatched on OP50, and L4s were treated with RNAi and sequestered for 48 h, then virgin day 2 adult males were dissected.

### Spermatid activation assay

Five virgin males were dissected in SM buffer containing BSA^35^. An equal volume of SM buffer containing 400 μg/ml Pronase (Millipore Sigma) was added, and spermatids were allowed to activate for 15 min. Images were manually scored, counting spermatids with pseudopodia as “activated.” As with sperm size analysis, L4 males were sequestered for 24 h before dissection, except in the case of *brf-1* RNAi, in which L4 males were isolated for 48 h.

### VIT-2::GFP imaging/quantification

Synchronous animals were washed from plates and fixed in 40% isopropanol for 2 h before a 30-min wash in PBS with 0.01% Triton-X100. Fixed animals were mounted and imaged on a Zeiss Axio Imager, and fluorescence of most proximal oocyte was quantified using ImageJ software (NIH). In order to ensure animals of the same developmental stage were quantified, only animals with exactly two embryos in utero were imaged. Fluorescence intensity per animal is equal to the sum of the corrected total cell fluorescence (CTCF) of both proximal oocytes.

### Developmental timing assay

Synchronous populations of L1 animals were dropped OP50, and beginning at 54 h post-drop scored for gravidity. Animals with uteruses containing at least one egg were considered to be gravid.

## Supplementary information

Supplementary Information 1.

Supplementary Information 2.

## Data Availability

All data are contained within the manuscript.
