# Supplementary material for: Roles for the RNA polymerase III regulator MAFR-1 in regulating sperm quality in Caenorhabditis elegans
Source: Sci Rep. 2020 Nov 9;10:19367. doi: 10.1038/s41598-020-76423-5 (PMC7652826; doi:10.1038/s41598-020-76423-5)
Supplement: Supplementary file 2 — Supplementary Information 2. [file 41598_2020_76423_MOESM2_ESM.pdf]

Roles for the RNA polymerase III regulator MAFR-1 in regulating sperm quality in *Caenorhabditis elegans*

Amy M. Hammerquist[1,2] and Sean P. Curran[1,2,3,*]

SUPPORTING INFORMATION

SUPPORTING INFORMATION

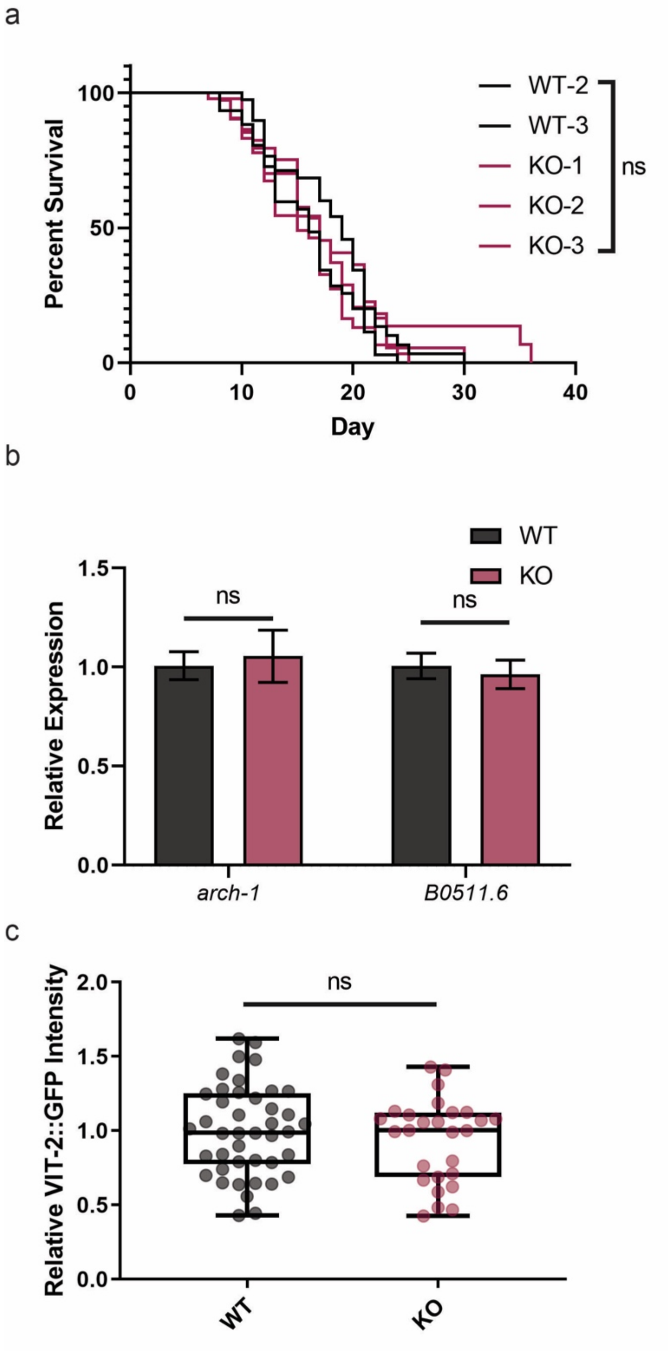

Supplemental Figure 1. Characterization of *mafr-1*(KO). (a) Lifespan survival assay of WT and *mafr-1* (KO) hermaphrodites. Individual lines represent populations of 50 worms. No significant difference was found by Log-rank (Mantel-Cox) test. (b) Quantitative PCR analysis of mRNA expression of *arch-1* and *B0511.6*, two genes that are expressed in an operon driven by *mafr-1*, in WT and *mafr-1* (KO) animals. No statistical difference found by Student's t-test (2-tailed). (c) Quantification of VIT-2 expression by fluorescence intensity of VIT-2::GFP fusion protein. No statistical difference found by Student's t-test (2-tailed).

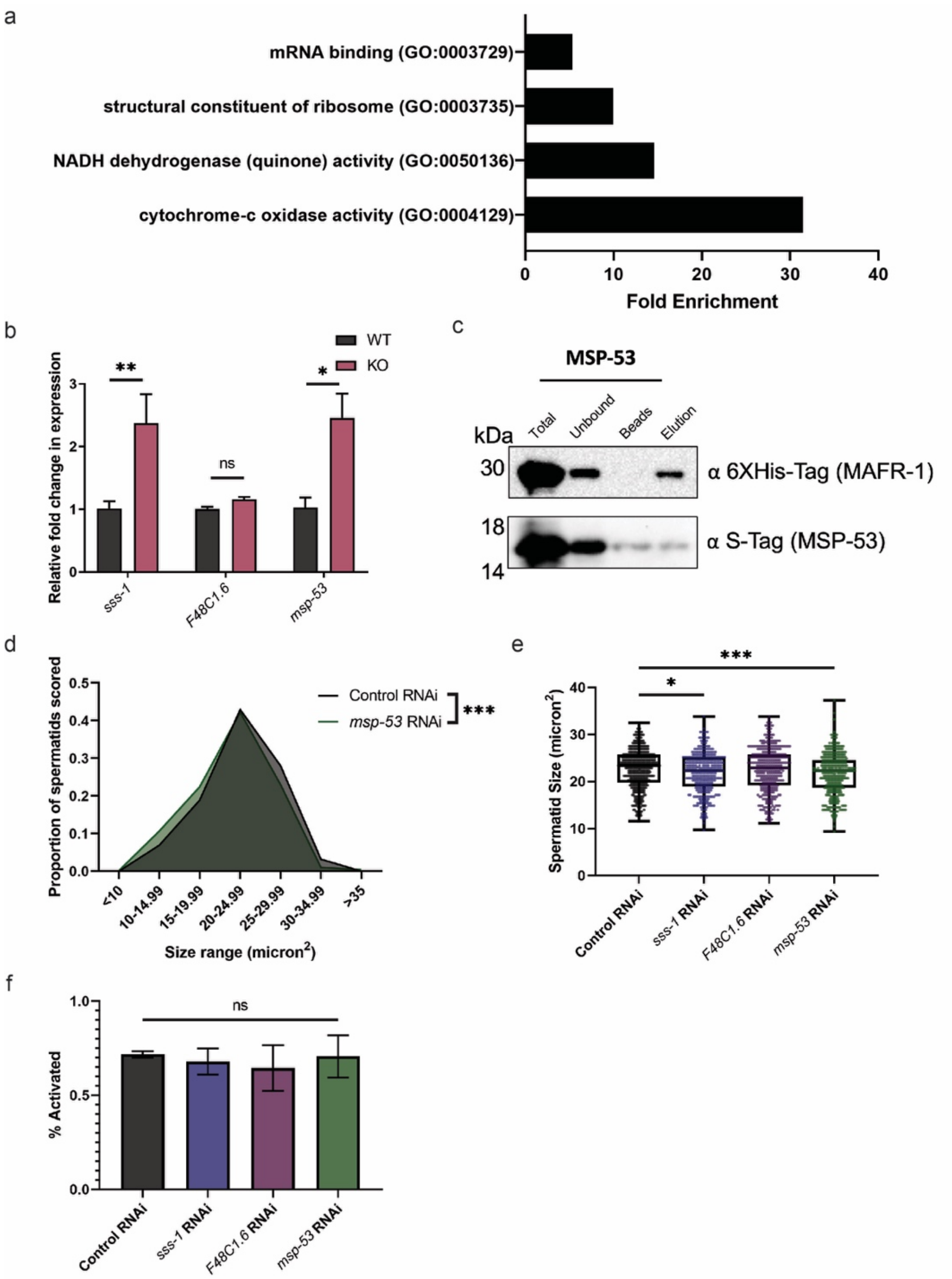

Supplemental Figure 2. Yeast two-hybrid identified diverse classes of putative interactors, including MSP-53. (a) Molecular function GO-term enrichment analysis of Y2H positive hits. (b) Quantitative PCR expression analysis of *sss-1*, *F48C1.6*, and *msp-53* in WT and *mafr-1* (KO) animals. (c) Western Blot analysis of Ni-NTA column co-purification of MAFR-1 and MSP-53. (d) Spermatid size in day 1 adult males following RNAi depletion of *msp-53*. Control RNAi are same data as depicted in **Fig. 2*c-d***. Experiment done in biological triplicate. See Supplemental Data Set for further statistical analysis. (e) Box-and-whisker plots displaying individual spermatid size values in day 1 adult males following RNAi depletion of *sss-1*, *F48C1.6*, and *msp-53*. The same data are depicted in **Fig. 2*c-d***, Fig. S2*d*. (f) *In vitro* Pronase activation of day 1 adult WT males following RNAi knockdown of *sss-1*, *F48C1.6*, and *msp-53*. Statistical comparisons made by Student's t-test (2-tailed). ns = no significance, * $p < 0.05$, ** $p < 0.01$, *** $p < 0.001$, **** $p < 0.0001$.

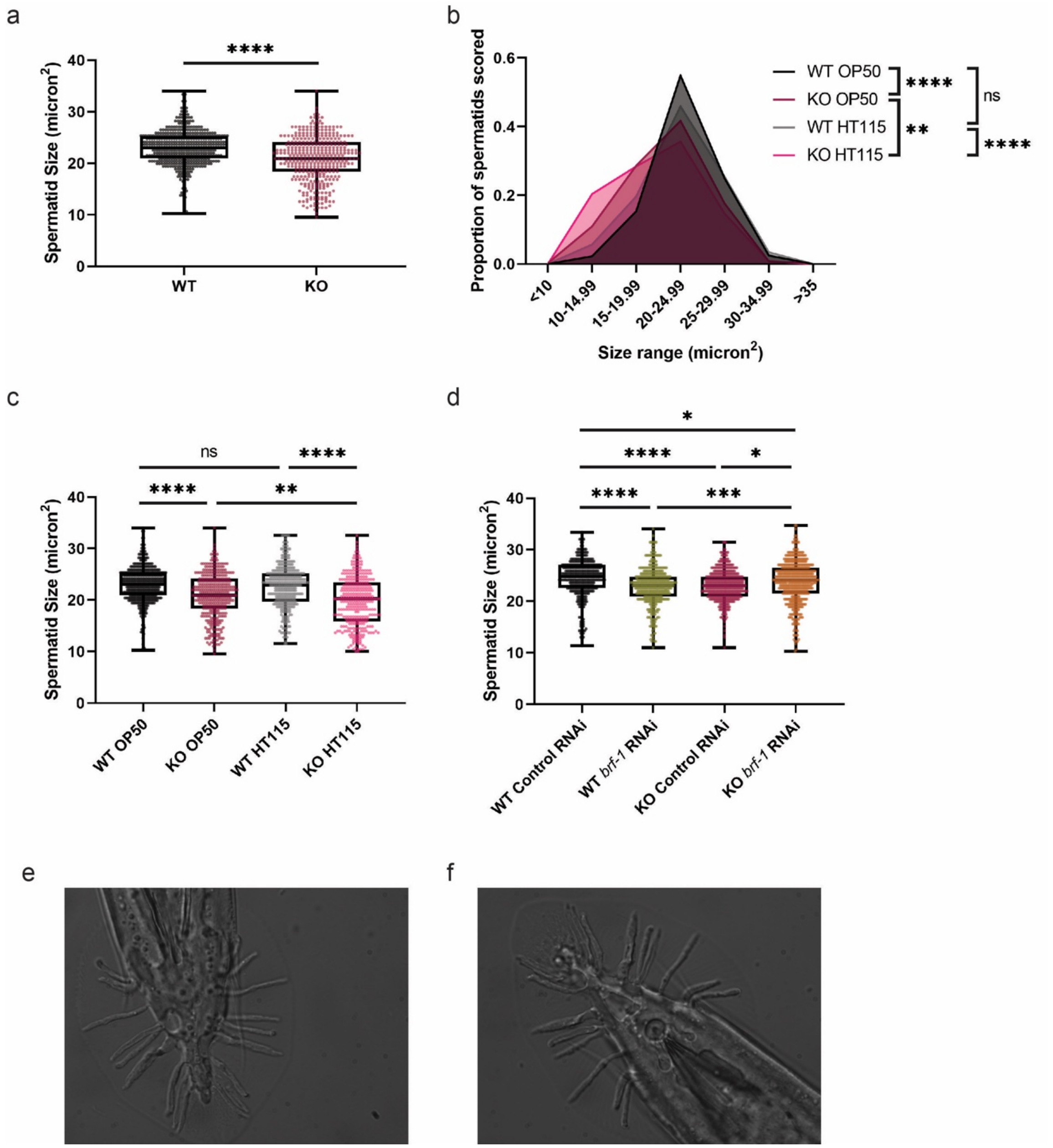

Supplemental Figure 3. *mafr-1* (KO) male sperm are smaller than WT and sensitive to diet. (a) Box-and-whisker plots displaying individual spermatid size values in day 1 adult WT and *mafr-1* (KO) males. The same data are depicted in **Fig. 3*A***. (b) Spermatid size in OP50- and HT115-raised WT and *mafr-1* (KO) males. See Supplemental Data Set for further statistical analysis. OP50 data are also represented in **Fig. 3*A***. (c) Box-and-whisker plots displaying individual spermatid size values in day 1 adult WT and *mafr-1* (KO) males fed OP50 and HT115 diets. The same data are depicted in Fig. S3*B*. (d) Box-and-whisker plots displaying individual spermatid size values in day 2 adult WT and *mafr-1* (KO) males following RNAi knockdown of *brf-1*. The same data are depicted in **Fig. 3*B***. (e-f) Representative images showing the tail rays of day 1 adult WT (e) and *mafr-1* (KO) (f) males.

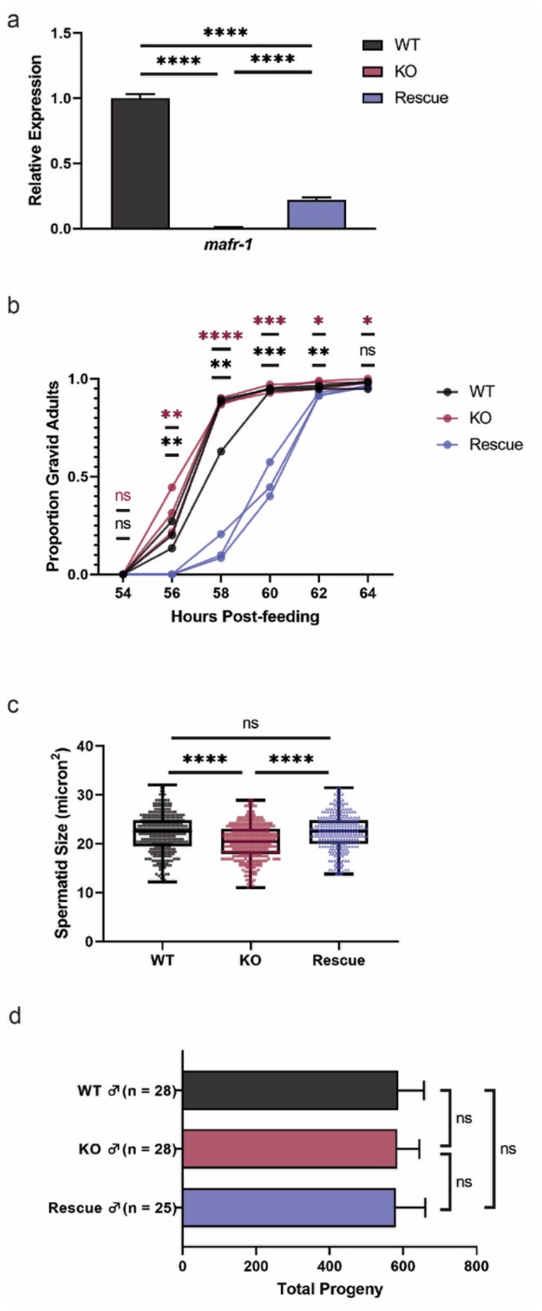

Supplemental Figure 4. Germline-specific rescue of *mafr-1* (KO) results in developmentally slow, but fertile animals. (a) Quantitative PCR analysis of *mafr-1* expression in WT, *mafr-1* (KO), and germline-rescued animals. (b) Developmental time course of WT, *mafr-1* (KO), and germline-specific rescue hermaphrodites. Data for WT and *mafr-1* (KO) are also depicted in **Fig. 1*b***. Black notations indicate comparisons between WT and Rescue, maroon notations indicate comparisons between *mafr-1* (KO) and Rescue. Reference **Fig. 1*b*** for comparisons between WT and *mafr-1* (KO). (c) Box-and-whisker plots displaying individual spermatid size values in day 1 adult WT, *mafr-1* (KO), and germline-specific rescue males fed OP50 and HT115 diets. The same data are depicted in **Fig. 4*a***. (d) Total brood size of WT, *mafr-1* (KO), and germline-specific rescue-mated WT hermaphrodites. Statistical comparisons made by Student's t-test (2-tailed). ns = no significance, * $p < 0.05$, ** $p < 0.01$, *** $p < 0.001$, **** $p < 0.0001$.

Supplemental Table 1. List of positive hits identified in yeast two-hybrid screen.

Sequence ID	Gene Identity	# Hits
K07E3.4	*C. elegans* unhcaracterized protein K07E3.4	1
D1054.11	*C. elegans* ULE-3	1
F25B5.5	*C. elegans* CDK5RAP1-like protein (F25B5.5)	1
C47B2.3	*C. elegans* TBA-2	1
C03C10.3	*C. elegans* RNR-2	1
T03F1.10	*C. elegans* CLEC-53	1
C27A2.6	*C. elegans* DSH-2	1
K08H10.7	*C. elegans* RDE-1	1
C34F6.2	*C elegans* COL-178	1
K02D7.3	*C. elegans* COL-101	1
F54C1.7	*C. elegans* PAT-10	1
ZK721.2	*C. elegans* UNC-27	1
cTel55X.1	*C. elegans* uncharacterized protein cTel55X.1	1
F32B6.6	*C. elegans* MSP-77	1
R13H9.4	*C. elegans* MSP-53/57	1
F32B6.5	*C elegans* SSS-1	1
Y71H2AM.20	*C. elegans* serine/threonine-protein phosphatase 2A activator Y71H2AM.20	1
Y71F9AM.6	*C. elegans* TRAP-1	1
F31E3.5	*C. elegans* EEF-1A.1	1
F57H12.1	*C. elegans* ARF-3	1
C30E10.4	*C elegans* GLY-20	1
F43D9.6	*C. elegans* URM-1	1
T03E6.7	*C. elegans* CPL-1	1
ZK945.2	*C. elegans* PAS-7	1
T26A5.7	*C. elegans* SET-1	1
F45E4.2	*C. elegans* PLP-1	1
Y48B6A.14	*C. elegans* HMG-1.1	1
MTCE.4	*C. elegans* NDFL-4	1
E04A4.7	*C. elegans* CYC-2.1	1
K07A12.3	*C. elegans* ASG-1	1
R10E11.8	*C. elegans* VHA-1	1
F46F11.5	*C. elegans* CHA-10	1
T20D3.5	*C. elegans* uhcharacterized protein T20D3.5	1
C14B9.10	*C. elegans* unhcaracterized protein C14B9.10	1
F53B6.4	*C. elegans* uncharacterized protein F53B6.4	1
T25B9.3	*C. elegans* uncharacterized protein T25B9.3	1

K03D3.5	*C. elegans* uncharacterized protein K03D3.5	1
R10E9.2	*C. elegans* uncharacterized protein R10E9.2	1
D1045.10	*C. elegans* uncharacterized protein D1054.10	1
F48C1.6	*C. elegans* uncharacterized protein F48C1.6	1
T24B8.2	*C. elegans* uncharacterized protein T24B8.2	1
C50D2.5	*C. elegans* uncharacterized protein C50D2.5	1
Y62H9A.2	*C. elegans* uncharacterized protein Y62H9A.2	1
T01C3.3	*C. elegans* uncharacterized protein T01C3.3	1
M28.10	*C. elegans* uncharacterized protein M28.10	1
F10B5.1	*C. elegans* RPL-10	2
F07D10.1	*C. elegans* RPL-11.2	1
C32E8.2	*C. elegans* RPL-13	1
M01F1.2	*C. elegans* RPL-16	1
E04A4.8	*C. elegans* RPL-20	1
C27A2.2	*C. elegans* RPL-22	1
C53H9.1	*C. elegans* RPL-27	1
R11D1.8	*C. elegans* RPL-28	1
F37C12.4	*C. elegans* RPL-36	2
B0041.4	*C. elegans* RPL-4	1
F54C9.5	*C. elegans* RPL-5	1
F56F3.5	*C. elegans* RPS-1	1
T01C3.6	*C. elegans* RPS-16	1
F53A3.3	*C. elegans* RPS-22	1
F28D1.7	*C. elegans* RPS-23	1
T05E11.1	*C. elegans* RPS-5	1
Y37E3.8	*C. elegans* uncharacterized protein Y37E3.8	1
